# The neuraminidases of MDCK grown human influenza A(H3N2) viruses isolated since 1994 can demonstrate receptor binding

**DOI:** 10.1186/s12985-015-0295-3

**Published:** 2015-04-22

**Authors:** Peter G Mohr, Yi-Mo Deng, Jennifer L McKimm-Breschkin

**Affiliations:** CSIRO Australian Animal Health Laboratory, Portarlington Road, Geelong, VIC 3219 Australia; WHO Collaborating Centre for Reference and Research on Influenza, 792 Elizabeth Street, Melbourne, VIC 3000 Australia; CSIRO Manufacturing, 343 Royal Parade, Parkville, VIC 3052 Australia

**Keywords:** Neuraminidase, Neuraminidase inhibitors, Oseltamivir, Zanamivir, Receptor binding, Influenza

## Abstract

**Background:**

The neuraminidases (NAs) of MDCK passaged human influenza A(H3N2) strains isolated since 2005 are reported to have dual functions of cleavage of sialic acid and receptor binding. NA agglutination of red blood cells (RBCs) can be inhibited by neuraminidase inhibitors (NAIs), thus distinguishing it from haemagglutinin (HA) binding. We wanted to know if viruses prior to 2005 can demonstrate this property.

**Methods:**

Pairs of influenza A(H3N2) isolates ranging from 1993–2008 passaged in parallel only in eggs or in MDCK cells were tested for inhibition of haemagglutination by various NAIs.

**Results:**

Only viruses isolated since 1994 and cultured in MDCK cells bound chicken RBCs solely through their NA. NAI inhibition of agglutination of turkey RBCs was seen for some, but not all of these same MDCK grown viruses. Efficacy of inhibition of enzyme activity and haemagglutination differed between NAIs. For many viruses lower concentrations of oseltamivir could inhibit agglutination compared to zanamivir, although they could both inhibit enzyme activity at comparable concentrations. An E119V mutation reduced sensitivity to oseltamivir and 4-aminoDANA for both the enzyme assay and inhibition of agglutination. Sequence analysis of the NAs and HAs of some paired viruses revealed mutations in the haemagglutinin of all egg passaged viruses. For many of the paired egg and MDCK cultured viruses we found no differences in their NA sequences by Sanger sequencing. However, deep sequencing of MDCK grown isolates revealed low levels of variant populations with mutations at either D151 or T148 in the NA, suggesting mutations at either site may be able to confer this property.

**Conclusions:**

The NA active site of MDCK cultured human influenza A(H3N2) viruses isolated since 1994 can express dual enzyme and receptor binding functions. Binding correlated with either D151 or T148 mutations. The catalytic and receptor binding sites do not appear to be structurally identical since relative concentrations of the NAIs to inhibit enzyme activity and agglutination differ.

**Electronic supplementary material:**

The online version of this article (doi:10.1186/s12985-015-0295-3) contains supplementary material, which is available to authorized users.

## Background

The influenza A haemagglutinin (HA) plays a major role during virus replication. It attaches the virus to the host cell by binding to sialic acid containing receptors and then initiates penetration [1,2]. The HAs from different viruses differ in their specificity and affinity of the receptors to which they bind. The neuraminidase (NA) is important for virus spread. The NA facilitates the release of newly assembled virions from host cells by cleaving sialic acids from cellular receptors. The role of influenza NAs is not limited to the removal of sialic acid from cellular glycoproteins, but also from viral glycoproteins to prevent newly assembled viruses from aggregating with each other [1,3]. The NA also facilitates dispersion through the mucus that coats ciliated epithelium during invasion of the human upper respiratory tract [4,5]. Additional NA functions have begun to emerge. For example, a potential role for NA assisting in viral morphology during influenza A(H10N7) replication has been proposed [6].

The avian influenza N9 subtype NA has been shown to have a non-catalytic second sialic acid binding site, distinct from the NA active site [7]. This site has a receptor binding function, demonstrated by binding to red blood cells (RBCs) and loss of function by mutagenesis [8]. There is a consensus sequence for this site which is highly conserved in avian influenza NAs [7]. The biological role of this second site is unclear. More recently the NAs of cell culture passaged human influenza A(H3N2) viruses have been reported to demonstrate receptor binding properties in addition to catalytic activity [9-11]. Unlike the N9 NA, receptor binding was reported to be in the NA active site.

Neuraminidase inhibitors (NAIs) are potent therapeutics specifically designed to bind the influenza NA active site [12] and therefore inhibit virion release. While based on the transition state analog of sialic acid, the NAIs bind in a different conformation in the NA active site compared to sialic acid in the receptor binding sites. Sialic acid binds in a chair conformation in both the HA and the N9 NA second binding site [7]. In contrast sialic acid and the NAIs bind in a twisted boat conformation in the NA active site [7,12]. Due to structural constraints, there is no evidence to date that the NAIs can bind in the receptor binding pocket of the HA or the second binding site of the N9 NA.

The necessity for balanced HA and NA functions in influenza viruses has been clearly demonstrated [13-15]. The virus requires sufficient HA activity to ensure attachment and enough NA activity for release from host cells. If these activities become unbalanced viral fitness is reduced, such as seen in the drug dependent NAI resistant viruses [15] with HA mutations resulting in a weak binding HA.

Since the emergence in 1968 of influenza A(H3N2) viruses with the HA derived from an avian strain, the virus has been constantly evolving as it adapts to infect humans. Since the early 1990s, the adaptation of H3N2 viruses in humans has caused a loss in their ability to be isolated from egg culture [16-19] suggesting a change in specificity or affinity of the HA for egg receptors. As a consequence the rate of successful H3N2 isolations of clinical specimens in eggs rather than mammalian cells is at least ten times lower [20]. In addition, many of the H3N2 clinical specimens isolated in mammalian cells do not agglutinate chicken red blood cells (CRBCs) or agglutinate poorly [11]. The loss of agglutination is attributed to the introduction of glycosylation sites in the region of the HA receptor binding site which appear to have more impact on binding to α2,3-linked sialic acids, like those found on chicken RBCs, than on α2,6-linked sialic acids, more predominant on turkey RBC (TRBC) [21,22].

Analysis by glycan arrays has not been able to demonstrate specific differences in egg and cell grown viruses and those which do or do not agglutinate CRBCs [11,23,24].

While it has been reported that the NAs of MDCK passaged human H3N2 viruses isolated since 2005 can demonstrate the receptor binding function, it is not known whether this property can be demonstrated by viruses prior to this period. There was significant antigenic drift of viruses between 1992 and 1997, which remained stable for the next 5 years, but significant changes occurred with the emergence in 2002 of the A/Fujian/411/2002-like virus [9] and subsequently in 2005 with the emergence of the A/Wisconsin/67/2005-like viruses.

In the present study we had pairs of influenza A(H3N2) isolates ranging from 1993–2008 which had been passaged in parallel either only in eggs or in MDCK cells. We demonstrated that NAIs inhibited the agglutination of CRBCs and TRBCs by only MDCK cultured influenza A(H3N2) viruses isolated since 1994 and 1996 respectively. While others have shown the acquisition of the NA receptor binding function correlates with a D151G/N mutation, deep sequencing did not detect a mutation at this site in all our isolates. However, those with only D151 had another variant population with a T148 mutation, a site reported to affect enzyme activity, zanamivir sensitivity, and possibly receptor binding [25]. Using a panel of different NAIs we showed differences in their ability to inhibit enzyme activity and receptor binding, suggesting sialic acid binds differently in the receptor binding and catalytic sites.

## Results

### Inhibition of haemagglutination by NAIs of egg or MDCK cultured viruses

Several groups have recently reported a receptor binding function associated with the NAs from H3N2 viruses isolated since 2005 after cell culture passaging [9-11]. NA receptor binding is discriminated from the HA binding as it is sensitive to inhibition by the NAIs, oseltamivir or zanamivir. Since significant antigenic changes in the NA occurred in 2002 and again in 2005 we were interested to determine if NAI inhibition of haemagglutination (NAI-HI) could also be demonstrated by pre 2005 H3N2 or other virus strains indicating this secondary function may be an optional property of other NAs.

We confirmed that CRBC agglutination of three cell passaged influenza A(H3N2) isolates from 2002 to 2006 was completely inhibited by oseltamivir or zanamivir at concentrations as low as 1 and 10 nM respectively (Table 1), thus confirming the role of their NAs in haemagglutination. Co-incubation of oseltamivir or zanamivir (0.01 nM to 100,000 nM) with seasonal influenza B, influenza A H1N1 or H1N2 viruses at 4 HAUs did not inhibit CRBC agglutination. The H1N2 viruses were a natural reassortant between the H1N1 and H3N2 strains which circulated briefly from 2001–3. Sequence comparisons of the NAs of H1N2 viruses isolated from England from the similar period to the one we tested show they contained almost identical NA sequences as those H3N2 viruses inhibited here by the NAIs. This does not rule out any of these other NAs also having receptor binding functions, as binding through HAs with higher avidity would prevent detection of inhibition of a weaker binding NA by the NAIs.

**Table 1:** **Minimum NAI concentration required to completely inhibit agglutination of chicken RBCs in the NAI-HI assay**

**Influenza Isolates**	**Passage** ^**a**^	**Oseltamivir** ^**b**^ ** (nM)**	**Zanamivir** ^**b**^ ** (nM)**
***Human***			
B/Perth/211/2001	MDCKx	-^c^	-
A/Mississippi/3/2001 H1N1	MDCKx	-	-
A/England/23/2002 H1N2	Ex/E1	-	-
A/England/23/2002 H1N2	MDCKx/MDCK1	-	-
A/Lipetsk/14/2002 H3N2	MDCKx	1-10	10-100
A/Okayama/23/2004 H3N2	MDCKx/MDCK1	10	100
A/Brisbane/22/2006 H3N2	MDCKx/MDCK1	1	10
***Swine and Avian***			
A/Swine/Nakorn Patham/2002 H3N2	Ex/E2	-	-
A/Duck/Selangor/659/2004 H3N2	Ex/E1	-	-
A/Duck/Tasmania/297/2006 H3N8	E2	-	-
A/Mallard/South Korea/12A/2006 H5N2	E4	-	-
A/Duck/Tasmania/277/2007 H7N2	E4	-	-
A/Chicken/Malacca/4905/2003 H9N2	Ex/E2	-	-

^a^E = egg, MDCK = Madin-Darby Canine Kidney, number of passages in culture condition stated or (x) unknown number of passages.

^b^NAI-HI assays were performed in duplicate with 4 HAUs of virus, a final concentration of 0.5% chicken RBCs and log_10_ dilutions of NAIs. Where consecutive drug dilutions showed partial then full inhibition a range between these two is given.

^c^No inhibition of haemagglutination (−) when tested with a maximum of 100,000 nM NAI.

To further investigate the extent of this phenomenon, influenza A(H3N2) isolates from swine and ducks as well as H3N8, H5N2, H7N2 and H9N2 viruses isolated from various avian sources were tested in the NAI-HI assay, but haemagglutination was not inhibited (Table 1). Thus haemagglutination was through their HAs and/or through the second discrete HB site described for avian influenza NAs [7], neither of which would be inhibited by NAIs.

To determine how far back this property could be demonstrated by human H3N2 strains, a selection of human H3N2 viruses was obtained that ranged in isolation date from 1993 to 2008. We obtained paired isolates that had been cultured in embryonated eggs or MDCKs independently, after the clinical specimens were collected (Additional file 1). We initially screened the viruses for inhibition by comparing the haemagglutination titres of the viruses in the presence or absence of 1 μM oseltamivir for both CRBC and TRBC. Oseltamivir did not inhibit haemagglutination by any of the egg-cultured viruses (results not shown) nor the MDCK cultured virus from 1993 (Table 2). In contrast, oseltamivir completely inhibited agglutination of CRBCs by MDCK passaged viruses isolated between 1994–2008 (Table 2). Therefore the ability of NAIs to inhibit haemagglutination of CRBCs by human H3N2 viruses dates back to at least 1994 (MDCK cultured virus was unable to be obtained for 1995).

**Table 2:** **Haemagglutination titres of MDCK cultured H3N2 viruses in presence and absence of 1 μM oseltamivir**

**Virus**	**CRBC**	**CRBC + Osel** ^**a**^	**Elution** ^**b**^	**TRBC**	**TRBC+ Osel** ^**a**^	**Elution** ^**b**^
A/Victoria/1/93	32-64	32-64	slow	64	64	slow
A/Victoria/4/94	2-4	<2	fast	8	8	med
A/Auckland/5/96	16	<2	fast	64	32	slow
A/Victoria/54/ 96	32	<2	slow	32	<2	slow
A/Auckland/19/96	16	<2	fast	32	16-32	slow
A/Victoria/6/97	8	<2	med	32	16	slow
A/Victoria/314/98	8	<2	fast	16	<2	med
A/Victoria/3/99	8	<2	slow	32	8	slow
A/Victoria/514/00	4-8	<2	slow	16	<2	slow
A/Perth/201/01	2	<2	med	16-32	16	slow
A/Perth/200/02	4	<2	fast	16	<2	slow
A/Christchurch/28/03	8	<2	med	16	<2	slow
A/Wellington/1/04	8	<2	fast	16	<2	med
A/Fukui/45/04	8	<2	med	16	<2	slow
A/Brisbane/3/05	2-4	<2	med	8	<2	slow
A/Victoria/503/06	4-8	<2	slow	4-8	<2	slow
A/Brisbane/10/07	4	<2	med	8-16	<2	slow
A/Brisbane/2/08	8-16	<2	slow	8-16	<2	slow

^a^Viruses were preincubated with oseltamivir at 4°C for 1 hour prior to the addition of 1% RBCs.

^b^Rate of elution of virus in the absence of inhibitor from RBC at 37°C, fast < 60 min, medium 1–3 hours, slow >3 hours.

There was however variation in the ability of oseltamivir to inhibit agglutination of MDCK grown viruses with TRBC. Generally the haemagglutination titres were higher for the TRBCs, suggesting either binding to different receptors, or with different affinity/avidity. However, when inhibition was seen, the concentration of NAI required for inhibition was often comparable to that for CRBC. Four of the viruses only showed marginal inhibition with a barely two-fold reduction in titre. Surprisingly, of three viruses tested from 1996 two were not inhibited, but a third virus was (A/Victoria/54/96).

We also compared the rate of virus elution from RBCs when the temperature was increased to 37°C to see if there was any correlation with the strength of binding and oseltamivir inhibition [9]. Elution will depend on both the affinity of the receptor binding and the NA activity. While elution varied from fast (<60 min) to slow (>3 hours) from CRBC nearly all the viruses eluted slowly from TRBC (>3 hours). However, there did not seem to be any correlation with agglutination inhibition by oseltamivir and rate of elution.

We then tested inhibition of agglutination of both CRBC and TRBC with serial log_10_ dilutions of oseltamivir and zanamivir, for the MDCK grown viruses from 1994 onwards. Agglutination of CRBC by all viruses was inhibited by both oseltamivir and zanamivir. Consistent with the initial screening with 1 μM oseltamivir, agglutination of TRBC of some of the viruses could not be inhibited either by oseltamivir or zanamivir at 1000 nM. Interestingly despite apparent differences in binding affinity between the CRBC and TRBC when agglutination of TRBCs was inhibited, the drug concentrations to inhibit agglutination were mostly comparable for each virus for both types of RBC. For many of the viruses the concentration of oseltamivir required to inhibit agglutination was up to 10-fold lower than for zanamivir (Tables 1 and 3). Again, despite differences in the RBC receptors this was observed with both CRBC and TRBCs.

**Table 3:** **NAI Inhibition of agglutination of MDCK cultured H3N2 viruses and NA 148 and 151 sequences**

**Name**	**CRBC osel** ^**a**^	**CRBC zan** ^**a**^	**TRBC osel**	**TRBC zan**	**Amino acid 151** ^**b**^	**Mutant percentage**	**Amino acid 148** ^**b**^	**Mutant percentage**	**Sequence coverage**
A/Victoria/4/94	1-10	10-100	>1000	>1000	-	-			-
A/Auckland/5/96	1-10	1-10	>1000	>1000	D		T/I	94%	256
A/Victoria/54/96	10	10	10	10	D/G	40%	T		560
A/Auckland/19/96	1-10	1-10	>1000	>1000	D		T/I	14%	230
A/Victoria/6/97	10	10-100	>1000	>1000	D/G	10%	T		500
A/Victoria/314/98	1-10	1-10	10	10	D		T/I	10%	250
A/Victoria/3/99	10	10-100	>1000	>1000	D/N	37%	T		300
A/Victoria/514/00	10	10-100	10	100	D/N	17%	T/I	12%	388
A/Perth/201/01	ND	ND	>1000	>1000	D/N	14%	T		535
A/Perth/200/02	10	10	10	10	D/N	6.5%	T/I	88%	1283
A/Christchurch/28/03	10	10	10	10-100	D/G	10%	T/K	24%	784
A/Wellington/1/04	10	10	10	10	D		I	100%	1005
A/Brisbane/3/05	10	10-100	10	10-100	D/N/G	27%/10%	T		630
A/Victoria/503/06	10	10-100	10	10-100	D/N/G	16%/11%	T		870
A/Brisbane/10/07	10	10	10	10	D/G	39%	T		1193
A/Brisbane/2/08	1-10	10-100	10	10-100	D/N	34%	T		1058

^**a**^Four HA units of virus were preincubated with log_10_ dilutions of oseltamivir (osel) or zanamivir (zan) for 60 min at 4°C prior to the addition of 1% RBCs. Titers are the minimum NAI concentration required to completely inhibit agglutination of chicken RBCs in the NAI-HI assay. Where consecutive drug dilutions showed partial then full inhibition a range between these two is given.

^b^Amino acids at position 151 and 148 based on Ion Torrent PGM deep sequencing.

### Sequence analysis of HA and NA genes

Others have reported a strong correlation between the emergence of a D151 NA mutation and the NA receptor binding function, and its inhibition by the NAIs [9-11]. Hence the HA and NA genes of seven pairs of isolates cultured in either eggs or MDCKs from 1996 to 2007 were sequenced by conventional Sanger sequencing (Genbank Accession Numbers Additional files 2, 3 and 4). Thirteen HA amino acid changes were detected in egg cultured viruses (Table 4, Additional file 3) with at least one amino acid change at known HA receptor binding or egg adaptation sites when compared to their MDCK cultured virus pair [24,26]. Changes at amino acid 194 were the most common with either L194I/P substitutions detected for four of the egg cultured viruses. This correlates with the findings of Lin et al. [9].

**Table 4:** **HA and NA changes between egg and MDCK cultured pairs of influenza A H3N2 viruses**

**Influenza A H3N2**	**Pass**	**NAI-HI** ^**b**^		**HA (H3)** ^**c**^										**NA (N2)**			
		**CRBC**	**TRBC**	**131**	**138** ^**d**^	**158**	**167**	**186** ^**e**^	**190** ^**d**^	**193** ^**e**^	**194** ^**d**^	**229** ^**e**^	**246**	**148**	**221**	**312**	**399**
**Consensus** ^**a**^				A/T	A	K/E	T	S/G	D	S	L	R	N	T	K/E	I	D/E
A/Auckland/19/1996	Egg	-								R							
	MDCK	+	-														
A/Auckland/5/1996	Egg	-									I						
	MDCK	+	-											I			
A/Victoria/3/1999	Egg	-									I	I					
	MDCK	+	+/−														
A/Perth/201/2001	Egg	-				N					I	I					
	MDCK	+	-	D			A										
A/Christchurch/28/2003	Egg	-			S			D	V								
	MDCK	+	+														
A/Brisbane/3/2005	Egg	-						V					K				
	MDCK	+	+														G
A/Brisbane/10/2007	Egg	-									P				K		
	MDCK	+	+													T	

^a^Consensus sequence based on alignment of HAs and NAs in this study.

^b^See Tables 2 and 3. As no egg grown viruses were inhibited with CRBC they were not tested with TRBC.

^c^H3 numbering [26].

^d^H3 receptor binding site amino acids [26].

^e^Egg adaptation amino acids of H3N2 viruses [24].

Initial alignment of the NA sequences (based on the amino acids as interpreted by the sequencing program) did not identify NA changes between the pairs of egg or MDCK cultured viruses of A/Auckland/19/1996, A/Victoria/3/1999, A/Perth/201/2001 and A/Christchurch/28/2003 viruses. There were only four obvious NA changes detected among all 14 viruses, with three (T148I, I312T and D399G) unique to MDCK cultured viruses. None of the four NA amino acid changes were in conserved regions or within the enzyme active site. The only egg culture NA change (E221K) was not unique with E and K being found in other viruses regardless of culture method (Additional file 4).

It was recently reported that T148I emerges in H3N2 NAs during MDCK passaging [25,27]. T148I was found to decrease zanamivir sensitivity but had no effect on oseltamivir sensitivity in an enzyme inhibition assay. Additionally T148I NAs had lower enzyme activity, but higher receptor binding, based on higher HA titers. In contrast we found no difference in either the HA titre, or in inhibition of haemagglutination by the A/Auckland/5/1996 MDCK cultured virus with the I148 compared to its counterpart A/Auckland/19/96 with T148.

In light of others reporting that the receptor binding function correlated with the emergence of a D151 mutation which they detected as a polymorphism originally by Sanger sequencing [9,28], we closely inspected each of the sequence traces for a possible dual peak.

Of the seven MDCK cultured H3N2 NAs we had sequenced several had dual peaks at position 151. However A/Auckland/19/1996 and A/Auckland/5/1996 had no trace of a population other than D151.

We therefore carried out deep sequencing (Ion Torrent PGM) to determine the amino acid at position 151 for the NAs of all of the MDCK cultured viruses from 1996–2008 (Table 3). Deep sequencing confirmed that there although there was variation for many of the viruses, there was no detectable variation at D151 for the two Auckland/96 viruses. Surprisingly two additional viruses also had no detectable amino acid other than D151, despite all having the receptor binding function.

Further deep sequencing analysis revealed that the four viruses with only D151 had populations with mutations of T148I (Table 3), varying from 10-100% of the population. Some of the other viruses with D151 mutations also had T148 mutations, including a T148K mutation. However, comparisons of the binding, inhibition and elution properties of the viruses with D151 and/or T148 mutations, showed that they varied within each group. The binding to TRBC could be inhibited for some, but not every virus with the same mutations (Tables 2 and 3). Furthermore there were differences in the rate of elution from CRBC within the groups (Table 2).

### Haemagglutination inhibition of oseltamivir resistant viruses

To further explore the relationship between inhibition of enzyme activity and receptor binding we investigated whether a mutation which leads to reduced oseltamivir sensitivity, but not reduced zanamivir sensitivity in the enzyme inhibition assay, affected their inhibition in the NAI-HI assay. We compared inhibition of agglutination by oseltamivir and zanamivir of A/Fukui/45/2004 H3N2 wild-type (wt) and E119V mutant virus pair [29] with both CRBC and TRBC. We also used peramivir which contains a hydrophobic side chain similar to oseltamivir, but its sensitivity in enzyme assays is not affected by the E119V mutation, and 4-amino DANA. Enzyme inhibition by 4-amino DANA is affected by the E119V mutation, due to altered interactions with its 4-amino group, the same as oseltamivir [12].

In the NAI enzyme inhibition assay the IC_50_ of oseltamivir for the E119V mutant was 150-fold higher than for the wild type (Table 5). In the NAI-HI assay, the E119V NA was also less sensitive to oseltamivir. It required 10-fold more oseltamivir to inhibit haemagglutination of the E119V virus than the wild type virus. The E119V mutation had no impact on zanamivir binding, either in the enzyme inhibition assay or the NAI-HI assay.

**Table 5:** **NAI inhibition of A/Fukui/45/2004 wild type and E119V enzyme activity and agglutination of RBCs**

	**Wild type**			**E119V**		
		**NAI-HI nM** ^**b**^			**NAI-HI nM** ^**b**^	
	**IC**_**50**_ **nM** ^**a**^	**CRBC**	**TRBC**	**IC**_**50**_ **nM** ^**a**^	**CRBC**	**TRBC**
**Oseltamivir**	1.7	1	1	260	10-100	10-100
**Zanamivir**	3.8	10	10	3.4	10	10
**Peramivir**	1.3	10	10	2.1	10	10
**4-Amino DANA**	970	1-10	1-10	25,100	1000	1000-10,000

^a^IC_50_s for oseltamivir, zanamivir and peramivir from Barrett et al. [44].

IC_50_ for 4-amino DANA mean of duplicate assays.

^b^NAI-HI assays were performed in duplicate with 4 HAUs of virus and a final concentration 0.5% RBCs.

In the enzyme inhibition assay the IC_50_ for peramivir was similar for both the wild type and the E119V mutant, and similar to zanamivir for the wild type (Table 5). Peramivir also inhibited haemagglutination of both the wild type and E119V mutant with similar efficacy, and comparable to zanamivir. As previously seen [12,15], inhibition of enzyme activity by 4-amino DANA was more than 100-fold less effective than by zanamivir (Table 5). Thus it was surprising that inhibition by 4-amino DANA in the NAI-HI assay was better than by zanamivir. The E119V mutation reduced binding of 4-amino DANA by >25 fold in the enzyme inhibition assay, consistent with the reduced binding of the V119 to the 4-amino group. It also required up to a 100 fold higher concentration of 4-amino DANA to inhibit the NAI-HI assay with the E119V mutant when compared to the wild type. Thus reduced sensitivity of both the enzyme activity and receptor binding of the E119V mutant to oseltamivir and 4-amino DANA supports the receptor binding function being part of the NA active site.

We initially observed among the MDCK cultured viruses listed in Tables 1 and 3 that for some viruses higher concentrations of zanamivir compared to oseltamivir were required to inhibit haemagglutination. This was also demonstrated by the A/Fukui/45/04 virus and its mutant, requiring 10-fold more zanamivir than oseltamivir to inhibit the haemagglutination of the wild type or E119V mutant virus. However, the amount of each drug required to inhibit enzyme activity was closer to a two-fold difference. The difference in efficacy of inhibition of enzyme activity and agglutination by 4-amino DANA was also unexpected. Hence, while all the NAIs tested inhibited both receptor binding and enzyme activity, potency for inhibiting enzyme activity did not correlate with potency for inhibiting receptor binding. Thus results suggest that while the two sialic acid binding sites are in the NA active site, they may not be identical. This would explain the differential inhibition of these two functions by the different NAIs.

### Inhibition of haemagglutination by DFSA inhibitors

We have recently described mechanism based inhibitors of the influenza NA, based on the substrate analogue 2,3 difluorosialic acid (DFSA) [30]. We also have both 4-amino and 4-guanidino modified derivates of these, with the 3-fluoro in either the axial (3Fax) or equatorial (3Feq) orientation. We tested inhibition of CRBC and TRBC binding with the A/Fukui/45/2004 MDCK grown virus. As seen for the 4-amino DANA, we found that despite having lower efficacy at inhibiting the enzyme activity (Table 6) one of the 4-amino derivatives was a more potent inhibitor of haemagglutination than the 4-guanidino derivatives. Thus, differences in potency of these different DFSAs for inhibition of enzyme activity and receptor binding further supports the hypothesis that while both functions are associated with the enzyme active site, the sialic acid may not be in identical positions. Hence interactions of the drugs with each site would differ.

**Table 6:** **Inhibition by Difluoro sialic acid inhibitors of A/Fukui/45/2004 agglutination of chicken RBCs and enzyme activity**

**Assay**	**DFSA nM** ^**c**^	**FaxAm DFSA** ^**c**^ **nM**	**FeqAm DFSA** ^**c**^ **nM**	**FaxGu DFSA** ^**c**^ **nM**	**FeqGu DFSA** ^**c**^ **nM**
^a^NAI-HI CRBC	100	100	10	100	100
TRBC	100	100	10	100	100
^b^Enzyme IC_50_	1380	4710	71	2006	25

^a^NAI^−^HI assays were performed in duplicate with 4 HAUs of virus, 0.5% RBCs and log_10_ dilutions of NAIs.

^b^IC_50_s from Kim et al. [30].

^c^DFSA = Difluoro sialic acid; FaxAm = 3 F axial 4-amino; FeqAm = 3 F equatorial 4-amino; FaxGu = 3 F axial 4-guanidino, FeqGu = 3 F equatorial 4-guanidino.

## Discussion

It has recently been demonstrated that the NAs of MDCK cultured human H3N2 viruses isolated since 2005 can have a dual function of both receptor binding and catalytic activity, both of which can be inhibited by the NAIs oseltamivir and zanamivir [9-11]. The receptor binding function is an NA dependent function, and is reported to be associated with mutations in the NA active site residue of D151G/N/A, rather than at a discrete second site as seen in the avian NAs [7]. While NA receptor binding has mainly been demonstrated by NAI inhibition of agglutination, inhibition of entry and infection of MDCK cells by the NAIs has also been demonstrated [11] confirming a relevant biological role for the NA receptor binding function.

There were significant antigenic changes with the emergence of the A/Fujian/411/2002-like virus in 2002 and again in 2005 with A/Wisconsin/67/2005 like viruses [9]. Hence we wanted to understand whether NA receptor binding could only be demonstrated by the NAs of recent isolates or whether earlier viruses could also demonstrate this property. For this purpose we obtained a panel of paired viruses grown only in cell culture or egg culture from 1993–2008. We tested their ability to agglutinate RBC and for inhibition of haemagglutination by the NAIs oseltamivir and zanamivir, indicative of NA mediated receptor binding.

Our results extended the observations of Lin et al. [9] showing that the NA receptor binding function could be demonstrated in the NAs of viruses as far back as 1994, and haemagglutination of MDCK-grown H3N2 viruses from 1994 to 2008 was inhibited by both zanamivir and oseltamivir. Haemagglutination by paired egg grown viruses was not inhibited, nor of the MDCK grown virus from 1993. How this receptor binding function is acquired through MDCK passaging cannot be determined, since the original clinical isolates are not available. However, despite differences in the NA sequences over the years, all of these N2 strains could demonstrate this function, suggesting it may be an optional function of human N2 NAs. It may also exist in earlier isolates, but be masked by a higher avidity HA being responsible for the receptor binding to CRBCs.

Although agglutination of CRBCs by all the post 1994 viruses was inhibited by the NAIs, inhibition by the NAIs of agglutination of TRBCs was only seen for some of the viruses, also observed by others [9,11]. CRBCs have higher levels of α2,3 linked sialic acids compared to α2,6 linked sialic acids, whereas TRBCs have higher levels of a2,6 linked sialic acids compared to CRBCs. Although these viruses clearly can bind both RBC receptors through their NA, their NAs may have different specificities or affinities for their target receptors. For these viruses their HAs may contribute more to binding TRBCs. Alternatively their NAs may be binding with much higher affinity to TRBC, hence they are not able to be inhibited.

Subsequent sequencing of the HAs of some of the virus pairs helped to establish that egg cultured viruses had mutations in the HA receptor binding site, which would correlate with the HAs of our egg grown viruses mediating haemagglutination for all the RBCs. It has also been shown that host specific glycosylation of the HA can affect its binding to target receptors [21], and it is possible that host cell glycosylation of the NA may also impact on its receptor binding. However there were no consistent differences in the HAs to account for the differences in the ability of the NAIs to inhibit TRBC agglutination by some but not all of the MDCK grown viruses.

Despite others reporting that D151G/N/A mutations in N2 NAs correlate with the receptor binding function [9-11] initial analysis of the sequences of the paired NAs of our MDCK and egg grown viruses showed no differences for some of the pairs. Others have seen polymorphisms in the codon for residue 151 by classical Sanger sequencing after culturing of H3N2 viruses in cells. This was confirmed by pyrosequencing [9,28]. Close examination of our Sanger sequence traces showed that although there was evidence of polymorphism of D151G and/or N for some viruses, surprisingly two viruses had no evidence of a mixed population. We therefore carried out Ion Torrent deep sequencing on the NAs of all the MDCK grown viruses from post 1996. This confirmed our Sanger sequencing results, but more surprisingly we found only D151 for an additional two viruses.

Further deep sequencing analysis revealed that these four D151 viruses all had a T148I mutation. Surprisingly, receptor binding was seen in virus stocks with as little as 10% of a mutant population. However, curiously viruses with the same D151 and/or T148 mutations did not demonstrate the same binding, inhibition and elution properties, e.g. TRBC binding by A/Auckland/5/96 (D151, 94% T148I) could not be inhibited by the NAIs, whereas TRBC binding by A/Wellington/1/04 (D151, 100% T148I) could be inhibited. This suggests that although mutations at D151 and/or T148 correlate with viruses demonstrating NA receptor binding, that additional secondary interactions of residues 151 and 148 may also be important for specificity of the receptor binding function.

Others have recently shown that a G147R mutation in the N1 NAs of pandemic H1N1, seasonal H1N1 and H5N1 viruses can also confer a receptor binding function [31,32]. Hence receptor binding may be an optional function of other NAs. As detection depends on having a poorly binding HA, this would make it generally difficult to detect.

We observed that while oseltamivir and zanamivir had similar IC_50_s in the enzyme inhibition assay, zanamivir was often less effective at inhibiting the haemagglutination by the NA. This was regardless of the D151/T148 mutations. Using a panel of NAIs with different chemical structures, we demonstrated differential efficacy between inhibition of enzyme activity and inhibition of haemagglutination for many of these NAIs. Some with a 4-amino group seemed to be more potent inhibitors than expected from their ability to inhibit enzyme activity.

While the normal function of the NA protein is to bind and cleave sialic acids on sialylated glycoproteins, to demonstrate receptor binding there must be subtle differences in the binding of sialic acid in the NA to preclude sialic acid cleavage. In trying to understand the relative locations of the catalytic and receptor binding sites Zhu et al. recently determined the structures of sialic acid in the N2 active site [10]. The sialic acid was in the distorted boat conformation. This is the same conformation normally seen in the NA catalytic site and would suggest interactions are the same for either receptor binding or enzyme activity. However, it is possible that the alternative chair-like conformation normally seen in receptor binding sites of the HA or the second binding site of the N9 NA [7] was too transient to detect. While they reacted the NA protein with glycans at 4°C prior to crystallization they still carried out crystallization at 22°C. Even for the N9 NA we only saw sialic acid in the second site when the complex was retained at 4°C, and not at 22°C [7]. Hence subtleties of the crystallization protocol may also be critical for detecting this second site in the N2 NA.

Our results showing the differential efficacy of several NAIs between inhibiting enzyme activity and receptor binding would in fact be consistent with the sialic acid binding in two different orientations and hence occupying subtly different parts of the enzyme active site for receptor binding and catalytic activity. Since higher concentrations of the 4-guanidino modified drugs are required to inhibit NA receptor binding, this suggests that the receptor binding site may overlap more where these drugs bind, than where those with a 4-amino substitution (oseltamivir and 4-amino DANA) bind.

The impact of some NA mutations on receptor binding has been studied using soluble NA [10]. However, there may also be an important contribution of avidity of multiple NAs or the NA and HA may act co-operatively to facilitate receptor binding. Thus to fully explore the roles of the D151 and T148 mutations and the HA in receptor binding, expression of native membrane anchored proteins is needed. The use of reverse genetics generated viruses will potentially lead to acquisition of NA mutations with virus amplification. Hence expression of the NA protein [33], with or without the HA in the baculovirus system, or the production of virus like particles [34] would be a valuable approach to broaden our understanding of receptor binding by the NA.

While it is well established that influenza viruses need to maintain a balance of the receptor binding function of the HA and the receptor destroying function of the NA, the additional property of receptor binding of the NA adds another level of complexity to maintaining this balance. Fortuitously the NAIs inhibit both functions.

## Conclusions

In conclusion, the N2 NAs of human influenza H3N2 viruses since 1994 cultured in MDCK cells can demonstrate dual catalytic and receptor binding functions. Both functions can be inhibited by a panel of NAIs, but there is not equal efficacy at inhibiting each function. This would be consistent with the sialic acid occupying subtly different parts of the enzyme active site for each function. Deep sequencing revealed mutations at amino acid D151 and/or T148 in all the NAs. It is also possible that host cell glycosylation of the NA may affect its receptor binding. With an increasing interest in targeting the HA for antiviral therapy [35-38], it is important to understand the secondary roles of the NA which may possibly compensate for the inhibition of the HA receptor binding function.

## Methods

### Viruses

Human influenza isolates were obtained from the WHO Collaborating Centre (Melbourne, Australia) except A/Lipetsk/14/2002 H3N2 [39], A/Mississippi/3/2001 H1N1 [40], B/Perth/211/2001 [41], A/Fukui/45/2004 wild type and E119V mutant and A/Okayama/23/2004 H3N2 [29]. Each virus was amplified in Madin-Darby canine kidney (MDCK) cells or in the allantoic cavity of embryonated chicken eggs (Golden Farms, Australia). Influenza A inactivated viruses of avian and swine origins were obtained from the OIE Avian Influenza Reference Laboratory (CSIRO, Australian Animal Health Laboratory AAHL, Australia).

### Chemicals and inhibitors

Zanamivir, peramivir and 4-aminoNeu5Ac2en (4-amino DANA) were provided by GlaxoSmithKline (Stevenage, UK). Oseltamivir carboxylate was kindly provided by Dr Keith Watson (Walter and Eliza Hall Institute, Australia). The difluoro sialic acids were kindly provided by Dr Stephen Withers [30]. The fluorescent substrate 4-methylumbelliferyl N-acetyl-α-D-neuraminic acid (MUNANA) was obtained from Carbosynth (UK).

### Haemagglutination assays

The haemagglutination titer of each virus was determined in U-bottomed microtiter plates with final concentrations of 0.5% chicken or 0.5% turkey RBCs (CSIRO AAHL and CSL, Australia, animal ethics approvals 815-2 and 891-1) according to standard protocols [42]. The virus titer was adjusted to 4 haemagglutination units (HAU) prior to use in neuraminidase inhibitor haemagglutination inhibition (NAI-HI) assays. NAIs and viruses were pre-incubated for 1 hour at 4°C before chilled RBCs were added. For the NAI-HI assay, NAIs were diluted in PBS with log_10_ drug dilutions ranging from 0.01 nM to 100,000 nM. Each virus stock was always tested simultaneously against both drugs in both CRBC and TRBC.

### NA enzyme inhibition assays

The MUNANA based fluorescent assay [43] was used for measuring inhibition of NA enzyme activity. Final concentrations in the assay were 50 mM sodium acetate pH 5.5, 5 mM CaCl_2_ and 100 μM MUNANA. NAIs were diluted in water, with dilutions ranging from 0.1 nM to 100,000 nM. Fluorescence was measured in a Fluoroskan Ascent Microplate Fluorometer (Thermo Scientific, USA) with 355 nM excitation and 460 nM emission filters. The IC_50_ was calculated as the concentration of inhibitor resulting in a 50% reduction in fluorescent units compared to the control.

### Sanger sequencing of viral genes

Viral RNA was extracted according to manufacturer’s instructions with the QIAamp Viral RNA Mini Kit (Qiagen, Germany). Reverse transcription of HA and NA genes was performed with SuperScript One-Step RT-PCR (Invitrogen, USA) using gene specific primers. The PCR amplicons were gel-purified (QIAquick, Qiagen) and sequenced by conventional Sanger sequencing (AAHL DSR Sequencing Lab, CSIRO, Australia). Full HA (KM978048 to KM978061) and NA (KM978062 to KM978075) open reading frames were submitted to GenBank. (Additional file 2).

### Deep sequencing with ion torrent PGM

Multi segment RT-PCR for whole HA/NA/MP was performed with MyTaq One-Step RT-PCR (Bioline, UK) using a set of cocktail primers (unpublished). A 200-bp DNA sequencing library was generated by fragmenting the PCR products followed by ligation to Ion Xpress Barcode Adapters using Ion Xpress Plus Fragment Library kit according to the manufacture’s instruction (Thermo Fisher, USA). The DNA library was loaded on Ion 314 Chip and run on Ion Torrent PGM (Thermo Fisher). Analysis of SNPs was performed using the variant Caller plugin supplied by the Torrent Server. A cut off of 5% was used for a SNP call if the sequence coverage of the site was more than 1000, and 10% if the coverage was below 1000.

## Additional files

Additional file 1:
**Passage histories of H3N2 viruses tested from 1993–2008.**
Additional file 2:
**Genbank Accession Numbers for HAs and NAs.**
Additional file 3:
**HA alignments of paired egg and MDCK cultured viruses.**
Additional file 4:
**NA alignments of paired egg and MDCK cultured viruses.**

## Abbreviations

HA: Haemagglutinin
HI: Haemagglutination inhibition
NA: Neuraminidase
NAI: Neuraminidase inhibitor
