# Supplementary material for: The neuraminidases of MDCK grown human influenza A(H3N2) viruses isolated since 1994 can demonstrate receptor binding
Source: Virol J. 2015 Apr 22;12:67. doi: 10.1186/s12985-015-0295-3 (PMC4409758; doi:10.1186/s12985-015-0295-3)
Supplement: Additional file 1: — Passage histories of H3N2 viruses tested from 1993–2008. [file 12985_2015_295_MOESM1_ESM.docx]

**Additional File 1: Passage histories of H3N2 viruses tested from 1993-2008.**

| **H3N2 Viruses** | **Cell/Egg passages^a^** | **Cell passages^b^** |
| --- | --- | --- |
| A/Victoria/1/1993 | E3/E2 | MK3/MD2/MD2 |
| A/Victoria/4/1994 | E3/E2 | x/MD3/MD6 |
| A/Shanghai/11/1995 | E5/E2 |  |
| A/South Africa/1147/1996 | x/E5/E2 |  |
| A/Auckland/19/1996 | CEK3/E2 | MDx/MD2/MD2 |
| A/Auckland/5/1996 | CEK1/E2/E1 | MDx/MD1/MD2 |
| A/Victoria/54/1996 | CEK3/E2/E1 | MD3 |
| A/Victoria/6/1997 | CEK1/E2/E2 | MDx/MD1/MD1 |
| A/Victoria/314/1998 | E3/E2 | MD2/MD3 |
| A/Victoria/3/1999 | E4/E3 | MD4/MD3 |
| A/Victoria/514/2000 | CEK1/E2/E1 | MDx/MD1/MD1 |
| A/Perth/201/2001 | E7 | MD3/MD2 |
| A/Perth/200/2002 | E5/E2 | MD4/MD4 |
| A/Christchurch/28/2003 | E7 | MD4/MD2 |
| A/Wellington/1/2004 | E3 | MDx/MD2/MD2 |
| A/Brisbane/3/2005 | E4/E1 | MDx/MD1/MD1 |
| A/Victoria/503/2006 | E3/E4 | MDx/MD1/MD2 |
| A/Brisbane/10/2007 | E2/E1 | MDx/MD4/ST1/MD2 |
| A/Brisbane/2/2008 | E4/E1 | MDx/MD1/MD3 |

Number of passages in each culture condition stated or (x) unknown number of passages.

^a^E = egg, CEK= chicken embryonic kidney,

^b^MK= monkey kidney, MD = Madin-Darby Canine Kidney, ST = MDCK-SIAT1.
