# Supplementary material for: The neuraminidases of MDCK grown human influenza A(H3N2) viruses isolated since 1994 can demonstrate receptor binding
Source: Virol J. 2015 Apr 22;12:67. doi: 10.1186/s12985-015-0295-3 (PMC4409758; doi:10.1186/s12985-015-0295-3)
Supplement: Additional file 2: — Genbank Accession Numbers for HAs and NAs. [file 12985_2015_295_MOESM2_ESM.docx]

**Additional File 2 Genbank Accession Numbers for HAs and NAs**

| **Virus** | **Culture** | **Accession Number** | **Accession Number** |
| --- | --- | --- | --- |
|  |  | **Hemagglutinin** | **Neuraminidase** |
| A/Auckland/5/1996 | Egg | KM978048 | KM978062 |
| A/Auckland/5/1996 | MDCK | KM978049 | KM978063 |
| A/Auckland/19/1996 | Egg | KM978050 | KM978064 |
| A/Auckland/19/1996 | MDCK | KM978051 | KM978065 |
| A/Victoria/3/1999 | Egg | KM978052 | KM978066 |
| A/Victoria/3/1999 | MDCK | KM978053 | KM978067 |
| A/Perth/201/2001 | Egg | KM978054 | KM978068 |
| A/Perth/201/2001 | MDCK | KM978055 | KM978069 |
| A/Christchurch/28/2003 | Egg | KM978056 | KM978070 |
| A/Christchurch/28/2003 | MDCK | KM978057 | KM978071 |
| A/Brisbane/3/2005 | Egg | KM978058 | KM978072 |
| A/Brisbane/3/2005 | MDCK | KM978059 | KM978073 |
| A/Brisbane/10/2007 | Egg | KM978060 | KM978074 |
| A/Brisbane/10/2007 | MDCK | KM978061 | KM978075 |
