# Supplementary material for: The neuraminidases of MDCK grown human influenza A(H3N2) viruses isolated since 1994 can demonstrate receptor binding
Source: Virol J. 2015 Apr 22;12:67. doi: 10.1186/s12985-015-0295-3 (PMC4409758; doi:10.1186/s12985-015-0295-3)
Supplement: Additional file 3: — HA alignments of paired egg and MDCK cultured viruses. [file 12985_2015_295_MOESM3_ESM.docx]

**Additional File 3: HA alignments of paired egg and MDCK cultured viruses.**

Auck1996AFH3 1 MKTIIALSYILCLVFAQKLPGNDNSTATLCLGHHAVPNGTLVKAITNDQIEVTNATELVQSSSTGRICDS

Auck1996MDH3 1 ......................................................................

Auck596AFH3 1 ...........................................T..........................

Auck596MDH3 1 ...........................................T..........................

Vic99AFH3 1 ....................................L......T................N.........

Vic99MDH3 1 ....................................L......T................N.........

Per01AFH3 1 ...........................................T.....................G....

Per01MDH3 1 ...........................................T.....................G....

Chris03AFH3 1 ........................................I..T.....................G....

Chris03MDH3 1 ........................................I..T.....................G....

Bris05AFH3 1 ..............................I.........I..T.....................G....

Bris05MDH3 1 ..............................I.........I..T.....................G....

Bris07AFH3 1 ...............T........................I..T.....................E....

Bris07MDH3 1 ...............T........................I..T.....................E....

Auck1996AFH3 71 PHRILDGKNCTLIDALLGDPHCDGFQNKEWDLFVERSKAYSDCYPYDVPDYASLRSLVASSGTLEFTNEG

Auck1996MDH3 71 ......................................................................

Auck596AFH3 71 ......................................................................

Auck596MDH3 71 ......................................................................

Vic99AFH3 71 ..Q....E.................................N........................N..S

Vic99MDH3 71 ..Q....E.................................N........................N..S

Per01AFH3 71 ..Q....E.................................N........................N..S

Per01MDH3 71 ..Q....E.................................N........................N..S

Chris03AFH3 71 ..Q....E............Q.......K............N........H...............N..S

Chris03MDH3 71 ..Q....E............Q.......K............N........H...............N..S

Bris05AFH3 71 ..Q....E............Q.......K............N...............I........N..S

Bris05MDH3 71 ..Q....E............Q.......K............N...............I........N..S

Bris07AFH3 71 ..Q....E............Q.......K............N........................N..S

Bris07MDH3 71 ..Q....E............Q.......K............N........................N..S

Auck1996AFH3 141 FNWTGVAQDGTSYACKRGSVKSFFSRLNWLHKLEYKYPALNVTMPNNDKFDKLYIWGVHHPSTDSDQTRL

Auck1996MDH3 141 ....................................................................S.

Auck596AFH3 141 ....................................................................SI

Auck596MDH3 141 ....................................................................S.

Vic99AFH3 141 ........N...S....R.I...........Q.K.............E....................SI

Vic99MDH3 141 ........N...S....R.I...........Q.K.............E....................S.

Per01AFH3 141 ........N...S....R.N...........Q.N.............E...................ISI

Per01MDH3 141 ......D.N...S....R.N...........Q.K........A....E...................IS.

Chris03AFH3 141 .D....T.N...SS...R.N..........TH.K.............E.............D...V.IS.

Chris03MDH3 141 .D....T.N...S....R.N..........TH.K.............E.............G.....IS.

Bris05AFH3 141 ......T.N...S....R.NN.........TH.KF............EE............V..N..IS.

Bris05MDH3 141 ......T.N...S....R.NN.........TH.KF............EE............G..N..IS.

Bris07AFH3 141 ......T.N...S..I.R.NN.........TH.KF............E.............G..N..IFP

Bris07MDH3 141 ......T.N...S..I.R.NN.........TH.KF............E.............G..N..IF.

Auck1996AFH3 211 YVQASGRVTVSTKRSQQTVIPNIGSRPWVRGISSRISIYWTIVKPGDILLINSTGNLIAPRGYFKIRSGK

Auck1996MDH3 211 ......................................................................

Auck596AFH3 211 ......................................................................

Auck596MDH3 211 ...............................V......................................

Vic99AFH3 211 .A.............................V..I...................................

Vic99MDH3 211 .A.............................V......................................

Per01AFH3 211 .A..P..........................V..I.................C.................

Per01MDH3 211 .A..P..........................V....................C.................

Chris03AFH3 211 .A.....I...................R..D.......................................

Chris03MDH3 211 .A.....I...................R..D.......................................

Bris05AFH3 211 .A....KI...................R..D.P..................K..................

Bris05MDH3 211 .A....KI...................R..D.P.....................................

Bris07AFH3 211 .A.....I...................R..N.P.....................................

Bris07MDH3 211 .A.....I...................R..N.P.....................................

Auck1996AFH3 281 SSIMRSDAPIGNCNSECITPNGSIPNDKPFQNVNRITYGACPRYVKQNTLKLATGMRNVPEKQTRGIFGA

Auck1996MDH3 281 ......................................................................

Auck596AFH3 281 ......................................................................

Auck596MDH3 281 ......................................................................

Vic99AFH3 281 ...........K..........................................................

Vic99MDH3 281 ...........K..........................................................

Per01AFH3 281 ........S..K..........................................................

Per01MDH3 281 ........S..K..........................................................

Chris03AFH3 281 ...........K..........................................................

Chris03MDH3 281 ...........K..........................................................

Bris05AFH3 281 ...........K..........................................................

Bris05MDH3 281 ...........K..........................................................

Bris07AFH3 281 ...........K..........................................................

Bris07MDH3 281 ...........K..........................................................

Auck1996AFH3 351 IAGFIENGWEGMVDGWYGFRHQNSEGTGQAADLKSTQAAINQINGKLNRLIEKTNEKFHQIEKEFSEVEG

Auck1996MDH3 351 ......................................................................

Auck596AFH3 351 .......................Y..............................................

Auck596MDH3 351 .......................Y..............................................

Vic99AFH3 351 ......................................................................

Vic99MDH3 351 ......................................................................

Per01AFH3 351 ......................................................................

Per01MDH3 351 ......................................................................

Chris03AFH3 351 ...................................................G..................

Chris03MDH3 351 ...................................................G..................

Bris05AFH3 351 ..........................I.............D..........G..................

Bris05MDH3 351 ..........................I.............D..........G..................

Bris07AFH3 351 ..........................I.............D..........G..................

Bris07MDH3 351 ..........................I.............D..........G..................

Auck1996AFH3 421 RIQDLEKYVEDTKIDLWSYNAELLVALENQHTIDLTDSEMNKLFERTRKQLRENAEDMGNGCFKIYHKCD

Auck1996MDH3 421 ......................................................................

Auck596AFH3 421 ......................................................................

Auck596MDH3 421 ......................................................................

Vic99AFH3 421 ......................................................................

Vic99MDH3 421 ......................................................................

Per01AFH3 421 ...............................................K......................

Per01MDH3 421 ...............................................K......................

Chris03AFH3 421 ...............................................K......................

Chris03MDH3 421 ...............................................K......................

Bris05AFH3 421 .............................................E.K......................

Bris05MDH3 421 .............................................E.K......................

Bris07AFH3 421 .............................................K.K......................

Bris07MDH3 421 .............................................K.K......................

Auck1996AFH3 491 NACIGSIRNGTYDHDVYRDEALNNRFQIKGVELKSGYKDWILWISFAISCFLLCVVLLGFIMWACQKGNI

Auck1996MDH3 491 ......................................................................

Auck596AFH3 491 ...............A......................................................

Auck596MDH3 491 ...............A......................................................

Vic99AFH3 491 ......................................................................

Vic99MDH3 491 ......................................................................

Per01AFH3 491 ......................................................................

Per01MDH3 491 ......................................................................

Chris03AFH3 491 .......................................................A..............

Chris03MDH3 491 .......................................................A..............

Bris05AFH3 491 .......................................................A..............

Bris05MDH3 491 .......................................................A..............

Bris07AFH3 491 .......................................................A..............

Bris07MDH3 491 .......................................................A..............

Auck1996AFH3 561 RCNICI

Auck1996MDH3 561 ......

Auck596AFH3 561 ......

Auck596MDH3 561 ......

Vic99AFH3 561 ......

Vic99MDH3 561 ......

Per01AFH3 561 ......

Per01MDH3 561 ......

Chris03AFH3 561 ......

Chris03MDH3 561 ......

Bris05AFH3 561 ......

Bris05MDH3 561 ......

Bris07AFH3 561 ......

Bris07MDH3 561 ......

**Abbreviations**

AFH3 = egg grown H3

MDH3 = MDCK grown H3

Auck1996 = A/Auckland/19/1996

Auck596 = A/Auckland/5/1996

Vic99 = A/Victoria/3/1999

Per01 = A/Perth/201/2001

Chris03 = A/Christchurch/28/2003

Bris05 = A/Brisbane/3/2005

Bris07 = A/Brisbane/10/2007
