# Supplementary material for: The neuraminidases of MDCK grown human influenza A(H3N2) viruses isolated since 1994 can demonstrate receptor binding
Source: Virol J. 2015 Apr 22;12:67. doi: 10.1186/s12985-015-0295-3 (PMC4409758; doi:10.1186/s12985-015-0295-3)
Supplement: Additional file 4: — NA alignments of paired egg and MDCK cultured viruses. [file 12985_2015_295_MOESM4_ESM.docx]

**Additional File 4: NA alignments of paired egg and MDCK cultured viruses.**

Auck1996AFN2 1 MNPNQKIITIGSVSLTIATICFLMQIAILVTTVTLHFKQYECNSPPNNQVMLCEPTIIERNITEIVYLTN

Auck1996MDN2 1 ......................................................................

Auck596AFN2 1 ..........................V...........................................

Auck596MDN2 1 ..........................V...........................................

Vic99AFN2 1 ......................................................................

Vic99MDN2 1 ......................................................................

Per01AFN2 1 ......................................................................

Per01MDN2 1 ......................................................................

Chris03AFN2 1 .......................................H..............................

Chris03MDN2 1 .......................................H..............................

Bris05AFN2 1 .................S....F......I...........FS...........................

Bris05MDN2 1 .................S....F......I...........FS...........................

Bris07AFN2 1 .................S....F......I...........F............................

Bris07MDN2 1 .................S....F......I...........F............................

Auck1996AFN2 71 TTIEKEICPKLAEYRNWSKPQCKITGFAPFSKDNSIRLSAGGDIWVTREPYVSCDPDKCYQFALGQGTTL

Auck1996MDN2 71 ......................................................................

Auck596AFN2 71 ......................................................................

Auck596MDN2 71 ......................................................................

Vic99AFN2 71 ......................N...............................................

Vic99MDN2 71 ......................N...............................................

Per01AFN2 71 ......................N...............................................

Per01MDN2 71 ......................N...............................................

Chris03AFN2 71 ...........V..........N...............................................

Chris03MDN2 71 ...........V..........N...............................................

Bris05AFN2 71 ......................D...............................................

Bris05MDN2 71 ......................D...............................................

Bris07AFN2 71 ......................D...............................................

Bris07MDN2 71 ......................D...............................................

Auck1996AFN2 141 NNRHSNDTVHDRTPYRTLLMNELGVPFHLGTKQVCIAWSSSSCHDGKAWLHVCVTGHDENATASFIYDGR

Auck1996MDN2 141 ......................................................................

Auck596AFN2 141 ......................................................................

Auck596MDN2 141 .......I..............................................................

Vic99AFN2 141 ...................................................................N..

Vic99MDN2 141 ...................................................................N..

Per01AFN2 141 ..G.....................................................D..........N..

Per01MDN2 141 ..G.....................................................D..........N..

Chris03AFN2 141 ..G............................R........................D.K........N..

Chris03MDN2 141 ..G............................R........................D.K........N..

Bris05AFN2 141 ..V.....................................................D.K........N..

Bris05MDN2 141 ..V.....................................................D.K........N..

Bris07AFN2 141 ..V......R...........................................I..D.K........N..

Bris07MDN2 141 ..V......R...........................................I..D.K........N..

Auck1996AFN2 211 LVDSIGSWSKKILRTQESECVCINGTCTVVMTDGSASGRADTKILFIEEGKIVHISPLSGSAQHVEECSC

Auck1996MDN2 211 ......................................................................

Auck596AFN2 211 ......................................................................

Auck596MDN2 211 ......................................................................

Vic99AFN2 211 ......................................K...............................

Vic99MDN2 211 ......................................K...............................

Per01AFN2 211 ......................................K...............T.S.............

Per01MDN2 211 ......................................K...............T.S.............

Chris03AFN2 211 ......................................K.................T.............

Chris03MDN2 211 ......................................K.................T.............

Bris05AFN2 211 .....V....E...........................K...............T.T.............

Bris05MDN2 211 .....V....E...........................K...............T.T.............

Bris07AFN2 211 .....V................................K...............T.T.............

Bris07MDN2 211 .....V....E...........................K...............T.T.............

Auck1996AFN2 281 YPRYSGVRCVCRDNWKGSNRPIVDINVKDYSIVSSYVCSGLVGDTPRKNDSSSSSHCLNPNNEEGGHGVK

Auck1996MDN2 281 ......................................................................

Auck596AFN2 281 ..Q...................................................................

Auck596MDN2 281 ..Q...................................................................

Vic99AFN2 281 ....P.................................................................

Vic99MDN2 281 ....P.................................................................

Per01AFN2 281 ....P................T....................................D...........

Per01MDN2 281 ....P................T....................................D...........

Chris03AFN2 281 ....P..............................................F......D...........

Chris03MDN2 281 ....P..............................................F......D...........

Bris05AFN2 281 ....P.....................I.....A.........................D...........

Bris05MDN2 281 ....P.....................I.....A.........................D...........

Bris07AFN2 281 ....P.....................I..H............................D...........

Bris07MDN2 281 ....P.....................I..H.T..........................D...........

Auck1996AFN2 351 GWAFDDGNDVWMGRTISEKFRSGYETFKVIGGWSKPNSKLQINRQVIVDRGNRSGYSGIFSVEGKSCINR

Auck1996MDN2 351 ......................................................................

Auck596AFN2 351 ...................L.............................................N....

Auck596MDN2 351 ...................L.............................................N....

Vic99AFN2 351 ...................L..........E...N.................S.................

Vic99MDN2 351 ...................L..........E...N.................S.................

Per01AFN2 351 ...................L..........E................................S......

Per01MDN2 351 ...................L..........E................................S......

Chris03AFN2 351 ...................L..........E.................E.....................

Chris03MDN2 351 ...................L..........E.................E.....................

Bris05AFN2 351 ...................L..........E...N...................................

Bris05MDN2 351 ...................L..........E...N.............G.....................

Bris07AFN2 351 ...................S.L........E...N.K.................................

Bris07MDN2 351 ...................S.L........E...N.K.................................

Auck1996AFN2 421 CFYVELIRGRKQETEVWWTSNSIVVFCGTSGTYGTGSWPDGADINLMPI

Auck1996MDN2 421 .................................................

Auck596AFN2 421 .................................................

Auck596MDN2 421 .................................................

Vic99AFN2 421 .........................L.......................

Vic99MDN2 421 .........................L.......................

Per01AFN2 421 ................L................................

Per01MDN2 421 ................L................................

Chris03AFN2 421 ...........E.....................................

Chris03MDN2 421 ...........E.....................................

Bris05AFN2 421 ...........E....L................................

Bris05MDN2 421 ...........E....L................................

Bris07AFN2 421 ...........E....L................................

Bris07MDN2 421 ...........E....L................................

**Abbreviations**

AFN2 = egg grown N2

MDN2 = MDCK grown N2

Auck1996 = A/Auckland/19/1996

Auck596 = A/Auckland/5/1996

Vic99 = A/Victoria/3/1999

Per01 = A/Perth/201/2001

Chris03 = A/Christchurch/28/2003

Bris05 = A/Brisbane/3/2005

Bris07 = A/Brisbane/10/2007
